# Innate and adaptive immunity in experimental glomerulonephritis: a pathfinder tale

**DOI:** 10.1007/s00467-016-3404-7

**Published:** 2016-05-11

**Authors:** Katharina Artinger, Alexander H. Kirsch, Ida Aringer, Foteini Moschovaki-Filippidou, Philipp Eller, Alexander R. Rosenkranz, Kathrin Eller

**Affiliations:** 1 Clinical Division of Nephrology, Department of Internal Medicine, Medical University of Graz, Auenbruggerplatz 27, 8036 Graz, Austria; 2 Intensive Care Unit, Department of Internal Medicine, Medical University of Graz, Graz, Austria

**Keywords:** Nephrotoxic serum nephritis, Immune system, T cell, Regulatory T-cell, Chemokines, Lymph node

## Abstract

The role of innate and adaptive immune cells in the experimental model of nephrotoxic serum nephritis (NTS) has been rigorously studied in recent years. The model is dependent on kidney-infiltrating T helper (TH) 17 and TH1 cells, which recruit neutrophils and macrophages, respectively, and cause sustained kidney inflammation. In a later phase of disease, regulatory T cells (Tregs) infiltrate the kidney in an attempt to limit disease activity. In the early stage of NTS, lymph node drainage plays an important role in disease initiation since dendritic cells present the antigen to T cells in the T cell zones of the draining lymph nodes. This results in the differentiation and proliferation of TH17 and TH1 cells. In this setting, immune regulatory cells (Tregs), namely, CCR7-expressing Tregs and mast cells (MCs), which are recruited by Tregs via the production of interleukin-9, exert their immunosuppressive capacity. Together, these two cell populations inhibit T cell differentiation and proliferation, thereby limiting disease activity by as yet unknown mechanisms. In contrast, the spleen plays no role in immune activation in NTS, but constitutes a place of extramedullary haematopoiesis. The complex interactions of immune cells in NTS are still under investigation and might ultimately lead to targeted therapies in glomerulonephritis.

## Innate and adaptive immune cells in the kidney in nephrotoxic serum nephritis

The nephrotoxic serum nephritis (NTS) model is a mouse model which is commonly used to study a type of immune complex-mediated, rapidly progressive glomerulonephritis (GN). It is induced by the injection of antibodies raised either in rabbits or sheep that are directed against the glomerular basement membrane (GBM). In the model used in our laboratory, a subcutaneous immunization against rabbit immunoglobulin G (IgG) prior to injection of the antiserum is needed to induce rapid-progressive GN within 7 to 14 days [1–3]. Other research groups using sheep anti-GBM antibody have reported that the immunization step is not required to induce a form of rapid-progressive GN [4, 5]. The so-called autologous phase of disease is characterized by nephrotic range albuminuria, a proliferative form of GN with crescent formation as well as infiltration of immune cells into the kidney [3]. The pathogenesis has been shown to be dependent on innate and adaptive immune cells as well as on the complement system. Kurts and coworkers recently published an excellent summary of the time course of kidney-infiltrating immune cells in NTS [6]. Gamma delta (γδ) T cells are the first cells to find their way into the kidney, attracting neutrophil granulocytes via the cytokine interleukin (IL)-17 [7]. Neutrophils, recruited via C-X-C motif chemokine ligand 1 (CXCL-1) [8], immediately infiltrate the kidney, mainly the glomeruli where they cause damage to glomerular cells. The absence of γδ T cells has been proven to protect mice from NTS [2]. Next, T helper (TH) 17 cells expressing CC chemokine receptor (CCR) 6 infiltrate the renal interstitium and glomeruli and in turn recruit neutrophils which infiltrate mainly the interstitium [9]. The recruitment of these neutrophils has been proven to be dependent on CXCL-5 rather than CXCL-1. CXCL-5 is induced in renal tubular epithelial cells by TH17 cells [8]. The prolonged infiltration of these adaptive immune cells seems to be dependent on dendritic cells (DCs) expressing CX3CR1 and CCR2 [10]. The latter also recruit TH1 cells, which lead to the infiltration of macrophages by the secretion of interferon-γ [11]. Depletion of either TH17 or TH1 cells significantly ameliorates the NTS phenotype in mice [5, 12]. DCs also recruit and activate effector T cells in the kidney by activation of the inflammasome and subsequent production of IL-1β and IL-18 [13]. In addition, activation of the inflammasome in macrophages by the P2X7 receptor increases disease activity [14].

Interestingly, not only pro-inflammatory cells infiltrate the kidney in NTS, but also regulatory immune cells which limit the on-going pro-inflammatory processes. In the early phase of disease, immature DCs recruit CCR6, expressing invariant natural killer T (iNKT) cells via the secretion of CXCL16 [15]. iNKT cells also have regulatory properties and suppress early infiltrating TH17 cells via the cytokines IL-4 and IL-10 [15]. TH17-specific STAT3-positive Tregs have recently been shown to infiltrate the kidney in the early phase of NTS and limit TH17 cell activation [16]. In the later phase of disease, regulatory T cells (Tregs) also infiltrate the kidney and limit TH1 activation by IL-10 secretion [6, 17, 18]. In early studies conducted in our laboratory, we did not detect early infiltration of Tregs into the kidneys [3, 19], but only infiltration in the prolonged phase of disease (Eller et al., unpublished observation). This difference might be explained by the different anti-GBM antibodies used as well as the need for immunization in our model.

Of note, the complement system activated by the IgG deposited in the glomeruli is also involved in the pathogenesis of NTS, especially in the model of accelerated NTS which needs pre-immunization against IgG. In the majority of studies, inhibition of the complement pathways resulted in an improvement of accelerated NTS [20–22]. In contrast, C1q deficiency—an early step of activation of the classical complement pathway—results in accelerated NTS disease activity probably due to a defect in the clearance of immune complexes and/or apoptotic cells from glomeruli [23].

Thus, both pro- and anti-inflammatory cell populations, including innate and adaptive immune cells, infiltrate the kidney during the course of NTS. Nevertheless, essential early immune regulation in NTS takes place in the secondary lymphoid organs, especially in draining lymph nodes. This is described in the following sections of this review.

## Immune regulation in the draining lymph node

The importance of the lymph node in the pathogenesis of NTS first became evident when CD4^+^ CD25^+^ Tregs were found to limit disease activity when transferred into mice prior to NTS induction. Interestingly, Tregs were not detectable in the kidney, but rather in secondary lymphoid organs and here mainly in the lymph nodes [19]. Evaluation of the role of CCR7 further contributed to a better understanding of the migration of Tregs in NTS. CCR7 is expressed not only on Tregs, but also on T cells and DCs [24]. By binding to their cognate CC chemokine ligands (CCL)-19 and -21, T cells and DCs are guided to the T cell zone of the lymph node, where DCs can present the antigen to T cells, which thereafter differentiate into effector T cells [24]. We have reported that CCR7 knock-out mice were more susceptible to NTS than controls due to a significant lower number of Tregs in the draining lymph nodes [3]. Interestingly, in our study the majority of Tregs were detected in the kidneys of CCR7 knock-out mice, but here they lost their ability to inhibit disease activity [3]. Thus, in the early phase of NTS, the correct localization of Tregs to the draining lymph nodes is paramount to an effective inhibition of disease progression.

MCs have also been shown to regulate NTS activity, mainly in the draining lymph node. MCs are known as main effector cells in allergic disease, but in recent years it has become evident that they have additional important roles in regulating the adaptive immune response by influencing the migration, proliferation and activation of B cells, T cells and DCs [25]. In the NTS model as well as in an anti-neutrophil cytoplasmic antibody (ANCA) vasculitis model, MCs have been found to inhibit the progression of kidney disease [26–28]. Interestingly, in a previous study we found that only a few MCs were present in the kidney, and those which were present were mainly located in the capsule; these MCs did not increase in number when NTS was induced [27]. In a subsequent study, we found that MCs were present in the draining lymph nodes and that they significantly increased in number during the course of NTS, mainly due to increased migration to the lymph node [29]. Targeting MCs by stabilizing agents has recently proven to limit ANCA vasculitis activity in the kidney in an experimental murine model and therefore makes the MC population an interesting new therapeutic target in GN [30].

Based on observations in skin transplant experiments [31], Lu and colleagues speculated that Tregs and MCs might interact with each other, also in NTS, and together inhibit disease activation in the draining lymph node. We proved that Tregs recruit MCs to the draining lymph node in the NTS model through Treg-derived cytokine IL-9 and that together these two cell populations form a microenvironment that inhibits effector T cells [29]. How MCs exert their anti-inflammatory capacity in NTS is still a focus of on-going research.

## Role of the spleen in NTS

It was long unclear whether the spleen plays a similar role as the draining lymph node in NTS. Since Tregs increased and homed not only to the draining lymph node but also to the spleen [3, 19], there was indirect evidence that the spleen might be of crucial importance in the immune regulation in NTS. Furthermore, spleen size and weight increased significantly during the course of NTS [32]. Data on the functional role of the spleen in human patients with GN are scarce. Case reports provide anecdotal evidence of a potential beneficial, but also harmful effect of splenectomy in patients with GN due to cryoglobulinaemia, IgA nephritis and connective tissue disease [33–35].

In a recent publication, splenectomized mice displayed comparable disease indices as sham-operated mice after NTS induction, indicating that the spleen has no direct role in the pathogenesis of NTS. Interestingly, significant differences in the haemoglobin levels were detected in splenectomized mice as compared to sham-operated controls. Obviously the increase in spleen size is mainly due to extramedullary haematopoiesis, which partly steps in for the significantly decreased haematopoiesis in the bone marrow observed in NTS [32].

## Proposed model of immune regulation in NTS

**Fig. 1:**
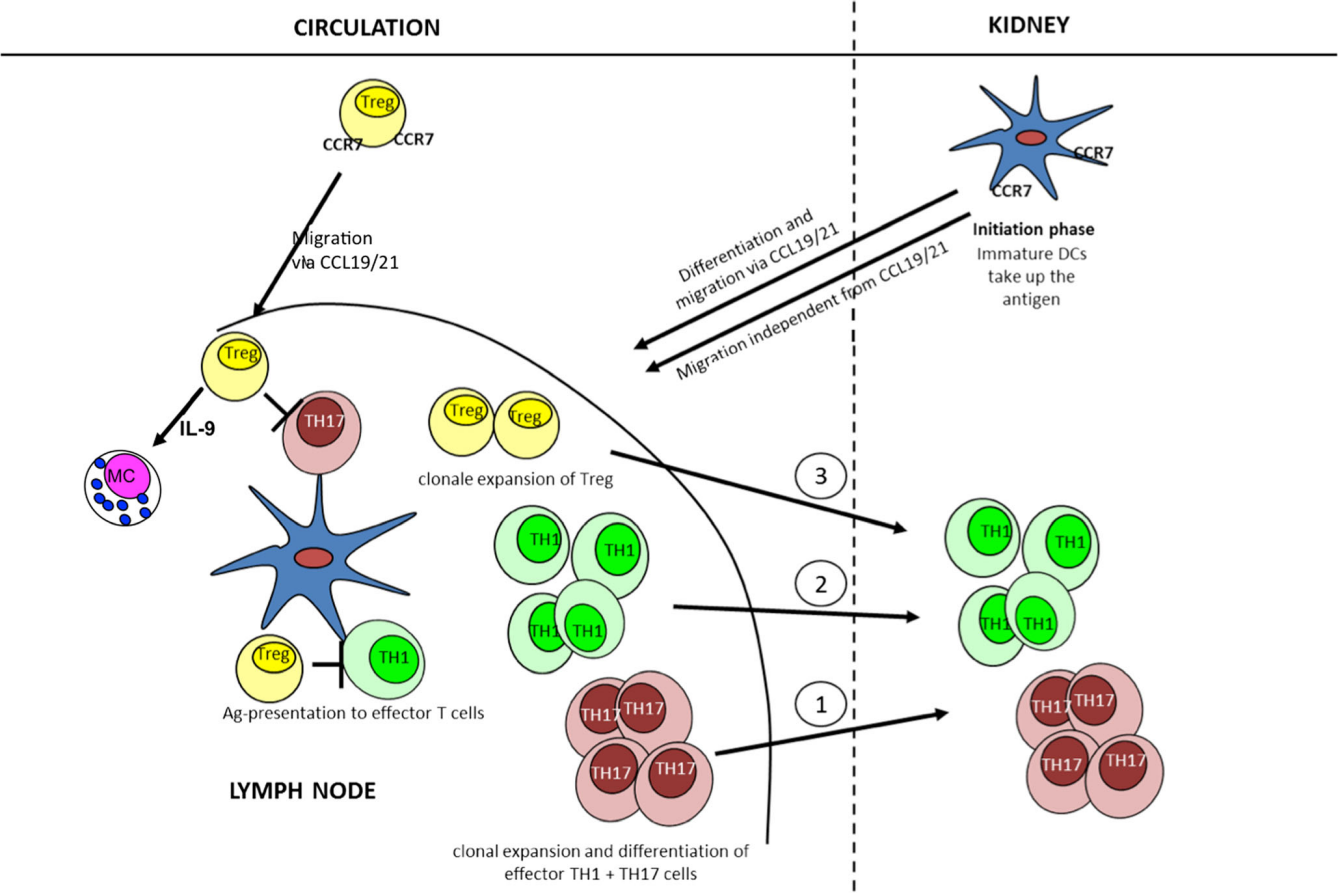
Proposed model of immune regulations in of nephrotoxic serum nephritis.* Ag* Antigen,* CCL* CC chemokine ligands,* CCR* CC chemokine receptor,* DC* dendritic cells,* IL* interleukin,* MCs* mast cells,* TH* T helper cells,* Treg* regulatory T cells

Based on the data of our group and other research groups, we propose the following model of immune regulation in NTS (Fig. 1). Two compartments are mainly involved in the immune regulation in NTS, namely, the draining lymph node and the kidney. We propose that DCs take up the antigen in the kidney and then migrate to the draining lymph node, where they present the antigen to T cells. To date, this mechanism has been proven for a tubular autoantigen [36], an endogenous kidney antigen, the Tamm–Horsfall protein [37], small molecular weight antigens that pass the glomerular filter [38] and a glomerular model autoantigen transgenically expressed in podocytes [39]. We propose that the migration of DCs to the draining lymph node in NTS is CCR7–CCL19/21 dependent, but we have also detected CCR7–CCL19/21-independent DC migration in NTS (Eller et al. unpublished data). In the draining lymph node, the antigen is presented to T cells, which differentiate and proliferate to effector TH cells. In NTS, TH1 and TH17 cells mediate disease activity [5, 12]. Both cell populations find their way into the kidney by the use of chemokine gradients as summarized by Kurts and coworkers [6]. The first TH cells entering the kidney in NTS are TH17 cells, which mainly recruit neutrophil granulocytes to the kidney via CXCL-5 [8]. Later in the course of the disease, TH1 cells migrate to the kidney and recruit macrophages. The inflammation is perpetuated by intrarenal DCs expressing CX3 chemokine receptor 1 (CX3CR-1) which process the antigen and keep stimulating effector T cells in the kidney [40] by inflammasome-dependent cytokines such as IL-1β [13]. Regulation of the immune response during the early phase takes place in the draining lymph node and is mediated by CCR7-expressing Treg cells, which effectively inhibit the differentiation and expansion of effector TH cells [3, 19]. This mechanism is partly mediated by a Treg-dependent recruitment of MCs to the draining lymph node via secretion of IL-9 and CXCL-1 [29]. How MCs exert their immunosuppressive capacities in the lymph node in NTS remains unclear at present. During the course of disease, Tregs also migrate to the kidney in an attempt to limit renal inflammation. It has been shown that both TH17-specific Tregs and CCR6-expressing Tregs are recruited to the kidney to limit intrarenal inflammation in NTS [9, 16–18].

## Conclusions

In recent past years different research groups have rigorously evaluated the various mechanisms of immune regulation and the migration of immune cells in the NTS model. Pro- and anti-inflammatory regulations interact closely in different compartments, namely, the draining lymph node and the kidney. Nevertheless, several questions in immune regulation remain unresolved, which argues for further research in the field to pave the way for targeted immune therapies of rapid progressive GN.
